# The next frontier in healthcare: perspectives and discussion on building trust and societal acceptance of digital humans in the essential framework of impact biomechanics

**DOI:** 10.3389/fbioe.2025.1693334

**Published:** 2025-12-19

**Authors:** Sebastien Roth

**Affiliations:** Laboratoire Interdisciplinaire Carnot de Bourgogne, UMR CNRS 6303 / Université de Technologie de Belfort-Montbéliard, Belfort, France

**Keywords:** numerical simulations, models validations, digital twins, impact biomechanics, human body models, societal acceptability, trust

## Abstract

The digital twin: this term present in many scientific fields corresponds to a virtual model of a physical object. Used in several framework on the physics, the digital twin must be fidelic to reality, by closely reproducing the behavior and interaction with its environment. However, it is necessary, before using such a tool, to ensure that it is indeed realistic. In the context of impact biomechanics, one of the objectives of which is to understand the mechanisms of injury occurrence and the tolerance threshold, and ultimately to allow optimization of protective systems that would reduce the risk of injury occurrence, the concept of the digital twin relates to the development of a numerical model that must be biofidelic before it can be used for the development of protection systems. This paper raises the question of the biofidelity and the protecting ability of a digital twin and how one can trust the digital procedure to develop protecting devices, without data coming from physical procedures.

## 1 Introduction

The digital twin: a term widely used across various domains today, refers to a virtual model of a physical object. Whether electronic, chemical, industrial, or mechanical, it is intended to faithfully represent reality when activated, by realistically reproducing the real-world. These tools are immensely valuable as they save time and money and also circumvent the constraints of certain procedures, notably ethical ones. In this context, it becomes possible to create multiple usage scenarios of a system using the digital twin and its dynamic nature, thus enabling evaluation of different system configurations or optimization thereof, without excessive expenditure of time or money.

This term “digital twin” is frequently employed within the scope of Industry 4.0 and the development of the factory/industry of the future. This particular case is extensively discussed in scientific literature, highlighting the applications and challenges of the digital twin (Pires et al., 2019), emphasizing the undeniable advantages of utilizing digital tools, such as the installation of an assembly line in the automotive industry as studied by Mendi (2022), which reports approximately a 6% increase in production efficiency and an 88% reduction in line downtime. However, it is important not to limit this concept solely to Industry 4.0, as scientific literature enumerates various domains that employ the concept of the digital twin. The literature review by Tao et al. (2022) clearly delineates different application domains, such as manufacturing, energy, environment, and agri-food industry. If the concept of the digital twin is “the hub of technologies of digital simulation of complex, multi-scale and multi-resolution physical phenome, enabling co-construction of the object of study by one or more humans,” as explained by Beal et al. (2021), then its application extends to the biomedical and biomedical engineering contexts as well.

Several recent articles in the literature define this concept and report on the opportunities and future directions in biomedical engineering research (National Academies of Sciences, Engineering, and Medicine, 2023; Grieves, 2023; Chakshu et al., 2024; Tang et al., 2024). In this domain, one of the subsections concerns impact biomechanics. Then, it is interesting to note that while the term “digital twin” (in the context of health) or “digital human” is relatively recent, its use in the context of impact biomechanics is much older and more commonly known as numerical modeling in biomechanics.

As defined by Zernicke et al. (2012), the prefix “bio” in biomechanics refers to research that deals with the understanding of molecules, cells, and organs. The term “mechanics” refers to the fundamental laws and theories of mechanical science, which ultimately apply to biological structures (Fung, 1993). The term impact biomechanics thus defines the study of the human body at different scales and the understanding of the physical phenomena observed when an object interacts with the human body at high velocity. One way of investigation into these phenomena, linked to the digital twin, is through numerical modeling, involving the creation of virtual models capable of simulating an impact. Thus, the first digital human or numerical biomechanical models appeared in the 1970s, and their use has evolved significantly in recent years due to the new possibilities offered by computing power. They are now capable of simulating complex impact configurations involving the human body and its environment and digitally replicating an experimental configuration.

As explained by Roth (2023), this type of simulation must address the following questions: how can the human body withstand an impact, and what types of injuries can occur? Based on the answers to these questions, how can protective systems be effectively developed? And finally, are the representations of the human body and its environment realistic enough to ensure the coherent and effective development of a protective system? Thus, replacing the real response of the human body with a “digital” response of the twin as a predictive tool for the development of a protection system requires confidence in the tool, and it is necessary, before its use, to ensure that it is realistic or predictive. In the context of impact biomechanics, one of the objectives is to understand the mechanisms of injury occurrence and the tolerance threshold; the concept of the digital twin/digital human relates to the development of a numerical model that must be “biofidelic” before it can be used for the development of protection systems. The question of biofidelity and how to ensure that the digital tool accurately represents reality is a central point and raises questions regarding the confidence and social acceptability of a “digital twin” in the development of a product intended for the public. This paper thus aims to establish the framework for the development of a digital human body model (digital twin), its validation (biofidelity), its use for injury prediction, and raises questions regarding the trust that one can place in a protection system developed from a digital procedure, which frees from real world experimental data.

## 2 Materials and methods

### 2.1 Preliminary statements: definitions and classical processes in impact biomechanics

The idea of the technology of “digital twin” was used at the beginning of the 90s by David Gelernter, who was one of the first to introduce the concept of “mirror world” using new technologies. As describe in his first sentences of the book, “this book describes an event that will happen someday soon: you will look into a computer screen and see reality” (Gelernter, 1993). The name ‘Digital Twin’ (DT) appeared in NASA’s technological roadmap in 2010 (Shafto et al., 2010).

The difference between digital model and simulation is not yet very clear, in the literature, even if recent literature tends to explain that “The virtual twin integrates human modeling and AI engines” (He et al., 2024). If the terms “numerical simulation” or “numerical modelling” are widely and classically used in the framework of numerical impact biomechanics, the term “digital twins” is also associated to biomechanics (Leal et al. 2023; Demir et al. 2023; Bahrami et al. 2025), leading to close definition of these concepts, and then ambiguous differences. We chose in this paper, to consider digital twins, digital model, or numerical simulation as equivalent, considering non-obvious differences in their definitions, in the framework of impact biomechanics.

In general, the development of a finite element model, within the context of biomechanics, involves several important stages. It begins with the digitization of an anatomical segment from medical images, followed by meshing and the implementation of material behavior laws. Boundary or initial conditions must also be implemented, with the possibility of including additional features such as interfaces or friction. Subsequently, to ensure the “biofidelity” of such models, an essential step involves the comparison of numerical results with experimental data that have been previously obtained. The numerical replication of the experimental setup then allows test/numerical data correlation and thus allows the validation of the finite element model. Following validation, the previously validated model is utilized for replicating real world case accidents: multiple cases are then reproduced with the model under different configurations (varying impact speeds, impact zones, types of impactors, etc.). These numerical data are then compared with clinical data from patients, and a statistical study provides numerical mechanical injury criteria and their threshold values. This classical process can be seen in several work of the literature (Willinger and Baumgartner, 2003; Kleiven 2007; Kleiven 2008; Roth et al., 2013; Bracq et al., 2019a). Following this process, the assessment of the protecting device can be conducted. As described by Roth (2023), the integration of a biomechanical model, encompassing injury thresholds and criteria, with a numerical model of a protective device for impact simulation yields insights into its protective efficacy. Should the numerical injury metric surpass the injury threshold, an injury is anticipated, needing redesign of the protective device. This methodology entails both numerical modeling and statistical examination of real-world trauma, from which injury metrics and thresholds are extrapolated. All the process raises then the question about the credibility of numerical simulation for a design of efficient protecting device purpose.

### 2.2 Benefits of digital processes

In the context of mechanical engineering, benefits of numerical simulations, are obvious. Classically, we list several advantages of its utilization, such as:

1. Reduced costs: the simulation allows reducing the costs linked to the manufacturing of prototypes and tests in laboratories.
2. Save time: numerical simulations allow accelerating the design process in reducing the time needed for the numerous iterations in the design. Results can be obtained widely quicker than for physical manufacturing and physical tests.
3. Detailed analysis: it allows investigating the mechanical behavior of a system at various scale. Indeed, mechanical parameters such as strain, stress, velocities can be analyzed with various time and dimensional scales.
4. Various scenarios: it offers the opportunity to investigate a wide panel of scenarios, configurations, and conditions of use, avoiding the need of manufacturing multiples prototypes for each scenarios.
5. Optimization: an optimization procedure can be coupled to the numerical simulation, allowing obtaining the best performance of a system, in minimizing the cost or the weight, keeping acceptable requirements.

In the framework of biomechanics, the role of numerical simulation is also crucial, providing essential knowledge in the understanding of processes and phenomenon. Indeed, the use of the simulation contributes to overcome difficulties to experimentally study the behavior of biological tissues. In that context, ethical issues can be pointed out: the use of biological tissues to conduct mechanical testing are fortunately governed by strict rules, that can prevent easy testing (Lopez-Valdes et al., 2024; Allen et al., 2017). In addition to this important latter point, experimental setups for testing biological tissues are complex to develop (various technics) and often costly (Navindaran et al., 2023; Costi et al., 2021); limitations that can be easily overcome by the use of digital twins. It can also provides information and data that cannot be observed or measured experimentally due to the speed of the phenomenon, especially for high speed loading configurations (ballistics or blast loadings) (Taddei et al., 2021; Roth, 2020; Bracq et al., 2019b). For biomedical design, it can also help to design medical devices such as prothetics, or surgical tools, by simulating their interaction with the human body (Akano, 2020; Ingrassia et al., 2013). The same kind of interaction between the human body with an external mechanical component can be explored for injury prevention, where the simulation can help to anticipate the injuries that can occur during an impact, and then in bringing conclusions about the relevance and efficiency of a protecting device to prevent injuries (Deck et al., 2003). Numerical tool can also have substantial benefit in rehabilitation strategies where the understanding of the phenomenon can lead to relevant treatments or surgery planning, for example, in the study of Marchetti et al., 2007, who proposed a computer-assisted surgical platform to simulate and evaluate the aesthetic consequences of skeletal and soft tissue adjustments in patients with dentoskeletal malocclusions.

Finally, as for standard mechanics, the numerical process can help to optimize the design of a component (protecting device, or prothetics, for example,) (Kowalczyk 2001; Rahman et al., 2014; Chang et al., 2012).

One of Aforementioned benefits concerns various configurations that can be studied using virtual framework. This point is interesting to investigate various environments in interaction with the model, but is also valid for the biomechanical model itself, where some numerical simulations can be conducted involving a specific anthropometry or morphology of the human body. Whereas experimental tests can not necessary provide post-mortem human subjects (PMHS) with a desired anthropometry (5th percentile female, or 95th percentile male subject), the concept of subject specific model can widely find its use and applications when using numerical tools (Karimi Dastgerdi et al., 2023; Schileo et al., 2007; Akrami et al., 2018), in order to derive mechanical parameters and tolerance thresholds dedicated to a given anthropometry.

Indeed, the change of model geometry to fit several morphologies can lead to general conclusions on a whole population and to study population-based clinical data, in taking into account population anthropometry variations. Numerical tools can then be used to make analysis considering the size and shape of a population, not only based on a 50th percentile male population, like it is generally the case in the context of impact biomechanics (Roth, 2023). The concept of a specific digital twin (known in the literature as subject specific biomechanical model) then becomes fully meaningful to obtain results and bring conclusion on a given subject (Sarvi and Luo, 2015).

Considering the scientific literature and applications which derive from human digital twins, their benefits cannot be questioned. However, we can raise some questionings concerning their performances, their improvements in a near future, challenges to overcome (He et al., 2024), and the way they are used, so that some concerns should be mentioned.

### 2.3 Questions and concerns about biomechanical numerical models

#### 2.3.1 Biofidelity of mechanical behavior law

When using FE simulation to simulate physical phenomenon, each step in the development of a numerical model can lead to potential errors or questions to raise. Indeed, the basis definition of such method being an “approximation” of the reality, all the mathematical formulations, physical assumptions, geometrical simplifications, mechanical or numerical uncertainties, can be combined to obtain wrong and non-biofidelic results. Thus, in that context, Oefner et al., 2021 proposed a methodological framework, in the context of biomechanical engineering, to identify clearly the difference steps of a FE simulation, and to provide key points, allowing a systematic analysis, in order to lead an appropriate simulation approach and unquestionable results. This methodology is based on the clear definition of various terms based on the work of Hicks et al., 2015, and on The American Society of Mechanical Engineers, 2007.

Each step of the process being a potential source of error, the different steps of a systematic flowchart and of a systematic process, should be strictly followed. Overall, this process systematically guides the user through the finite element simulation process, ensuring each step is meticulously planned, executed, and reviewed to produce reliable, accurate and unquestionable results.

As explained previously, even if each step of the process is accurately conducted, and can then minimize the appearance of an error, the concept of modelling itself, can bring some uncertainties or errors in the calculations, particularly when the numerical expert make a modelling choice leading to a “user dependent choice”. Especially, in the context of numerical simulation in biomechanics, where lots of approximation are performed on the human tissues behavior, on the interaction between physical, chemical, biological phenomenon, mechanobiology, on the interaction between phenomenon at different scales, the sources of modelling errors are numerous. A classical example of this complexity lies in the choice of the constitutive law. For example, Holzapfel and Ogden, 2025 wrote recently a complete review on nonlinear constitutive models for the mechanics of soft biological tissues, and various elastic or inelastic behavior such as viscoelasticity, damage, poroviscoelasticity, and also highlighted the importance of the microstructure within the tissues. The same author also provided very interesting openings and directions for further research including artificial intelligence and specifically deep learning (Holzapfel et al., 2021), pointing out the significative improvements that could be conducted to enhance actual mechanical modelling of soft tissues, in using mechanical tests, histological analysis and image processing to train a model for arteries behavior prediction. In the same framework of soft tissue biomechanics, deep learning is a concept also used to infer material properties of abdominal aortic aneurysms or the aorta for example, (Joldes et al., 2017; Liu et al., 2019). Precise states of the art and perspective on these technics of artificial intelligence can be found in the work of Donmazov et al. (2024), Fu et al. (2025) or Wu et al. (2024). Although theses new recent technics can have a high potential for the definition of biofidelic soft tissues, including micro-macro relationships for modelling at various scale, they can however, bring some new uncertainties in the evaluation of mechanical parameters and in the choice of the constitutive laws.

In addition to the complexity of the behavior law, the user dependence choice also lies in the objective and the configuration of the simulation which need to be investigated, and which are also directly linked to the choice of the behavior law. In terms of objective, does the given simulation necessarily requires the implementation of a rupture criteria of the soft tissue, or could a more simple law be sufficient? In terms of configuration, does the impact involve an active or passive situation of the soft tissues (for example, muscles which can alter their properties when activated) (Ehret et al., 2011; Holzapfel and Ogden, 2018). Does the impact involve a high strain rate needed to be taken into account in the constitutive law (Tran and Tsai, 2023; Hu and Desai, 2004)? These questions raise relevant questions about the biofidelity.

#### 2.3.2 Credibility of models: verification and validation of the numerical process

Finite element analysis (FEA) has emerged as an essential method for biomechanical research. Although there are various recommendations aimed at standardizing research methods and reporting results, many challenges still remain in the use of computational models in the framework of biomechanics. These challenges concern the way to check and validate models, as well as the assessment of a model, as wisely explained and described for example, by Oefner et al. (2021) in his methodology dedicated to orthopedic and trauma biomechanics, and based on previous verification process [ASME VV-10-2006]. Indeed, this study presents a detailed checklist of keypoints that need to be clearly examined, at a numerical level, such as the mesh quality and density, the uncertainty quantification, the sensitivity analysis, the identification of inputs parameters, etc… including a peer review process named “four-eyes principles”, consisting of verifying the whole procedure to avoid possible errors.

Finally, the use of numerical models in biomechanics has, for several decades, already demonstrated its utility and benefits both in advancing the understanding of trauma mechanisms and in contributing to the improvement of human body protection systems (this established relevance is by no means questioned in the present work). But taking into account the various sources of uncertainty inherent to the development and validation of a numerical model, both at numerical and mechanical level, is of interest, and it is reasonable to question the reliability and practical applicability of a biomechanical numerical model as a foundational tool for the design of a protective device.

## 3 Discussion

### 3.1 Trust and acceptability of “models” for product development

Considering all the previous paragraphs of Section 3, and all the uncertainties that can be involved in the development of a numerical model, including the validation process, questions are allowed when considering the efficiency and the use of a biomechanical numerical model, as a basis for the development of a protecting device.

Even if validated, how can one trust a model?

Even if validated, how can the society accept to use a product developed only through a numerical-based method?

In order to assess the concept of trust and acceptability of numerical models in the literature, a PRISMA analysis has been conducted. “Preferred Reporting Items for Systematic Reviews and Meta-Analyses” (PRISMA) Statement (Moher et al., 2009), is a well-known method, widely used to conduct systematic review, and which allow pointing out the review strategies of researchers which brought them to consider some scientific papers or not, and to highlight the way they can analyze all the scientific meta-data to reach to their statistical conclusion.

The review was guided and based on recommendations as follow:

Objectives: This study presents an analysis on the use of human body digital model to be used for numerical analysis of protecting systems, for protecting device development.

Eligibility criteria: Studies that include some statements related to trust and acceptability of digital model in a process that aim to design new physical products (in our case protecting device) are included in the review. After analysis of the articles provided in the database, those with no link to the present research were excluded.

Information sources: Two scientific database including scientific journals dealing with digital twins/digital model for healthcare were considered in July 2025: Web of Knowledge and Google Scholar.

Data management: Four keywords were used to highlight the studies to be considered for analysis: “Trust” AND “acceptability” AND “digital model”, AND “healthcare”.

Bias(es): These keywords encompass broad concepts that extend beyond the scope of impact biomechanics; we will focus solely on articles addressing impacts within the field of biomechanics. Thus the number of articles provided by the databases may be larger than the number of articles focusing on the specific content of the present article.

Data synthesis: The keywords were intentionally selected with broad scope so as to avoid excluding potentially relevant studies. Statistical conclusion of the meta-data research are obvious and if the researchs on the database provide 35 articles on Web Of Science database, and 1,010 articles on Google Scholar database, none of these articles interest in the specific topic of digital models for biomechanics.

The concept seems then very poorly explored in the literature. However, if we extend the concept of digital twin or digital human to a more general concept, such as “artificial intelligence” or “algorithms-based methods”, and for global medical applications, research can find more papers dealing with trust and acceptability, which generally concern data-driven methods for clinical decisions. Lots of these papers can be found in the systematic review conducted by Evans et al. (2024). Another interesting article, but which also falls outside the precise scope of “impact biomechanics” of the present article, is the article of Gillespie et al. (2025), which precisely deals with the trust of AI. The use of digital model for impact biomechanics can potentially be included in a AI framework (Li et al., 2022; David et al., 2023), questions about trust of biomechanical models may fall within Gillespie’s framework, showing that half of the population are wary about trusting AI, even if a large majority accept its use.

If a specific focus is made on biomechanical numerical models for impact, It would be of interest to precisely conduct a survey to investigate the trust and acceptability of algotihmic-based process for medical purpose and applications to human protection which derive from these numerical process.

### 3.2 Experimental results for validation purpose can also be questioned

Although previous paragraphs highlighted a number of uncertainties that can be involved in a numerical model and that can lead to non-biofidelic results, it is also important to explain how experimental results can also be potentially biased, and how these results can provide bad references for validation purpose, since mechanical testing standard and protocols have not been established for all anatomical segments, as demonstrated in studies of the literature (Genter et al., 2023; Newell et al., 2020, or Costi et al., 2021).

We highlight hereafter several key points that may challenge the perceived absolute and unquestionable nature of mechanical testing in biomechanical engineering.

For a given population, some significant differences exist both in terms of morphology, and in mechanical properties of anatomical segments. These classical differences are widely investigated in scientific literature:

In terms of morphology, the study of Chen et al. (2021) highlight significant difference in the Taiwanese population, for several dimensional characteristics such as body weight, height. With a specific focus on the elderly, Canaan Rezende et al. (2015), also pointed out the same kind of conclusion about anthropometry variability. With the illustration of two CT-scans, Roth et al. (2013) also exhibited morphological differences.

This point can be illustrated by the concept of “percentile”, which consist in categorizing the human body as a function of body dimensions (height, head circumference, weight, etc…), in 5th percentile, 50th percentile or 95th percentile category (NASA, 2025). Then, these points illustrate differences that can occur for experimental results in biomechanics, and which could question the data used for numerical validation.

In addition, in terms of mechanical characterization, the literature also lists a number of studies which illustrate the mechanical behavior of a biological tissue, with a range of responses, for various anatomical segments which have been tested mechanically, with given experimental devices (Li et al., 2013; Hou et al., 2025].

In addition, it can also be noticed the obvious difference between men and women, both in terms of morphology and mechanic behavior of tissues as investigated by Roth (2023).

Finally, combining the ranges of responses of the human body linked to differences in terms of anthropometries and in terms of mechanical properties, experimental corridors can then be defined: all the responses of the human body of a given population are within the experimental corridor.

These corridors are interesting because they consider a given population. Then, in terms of numerical model, the numerical response of a 5th, a 50th or a 95th percentile model should be within the corridor to be considered as a biofidelic validated model (Roth et al., 2013; Roth et al., 2010).

**FIGURE 1:**
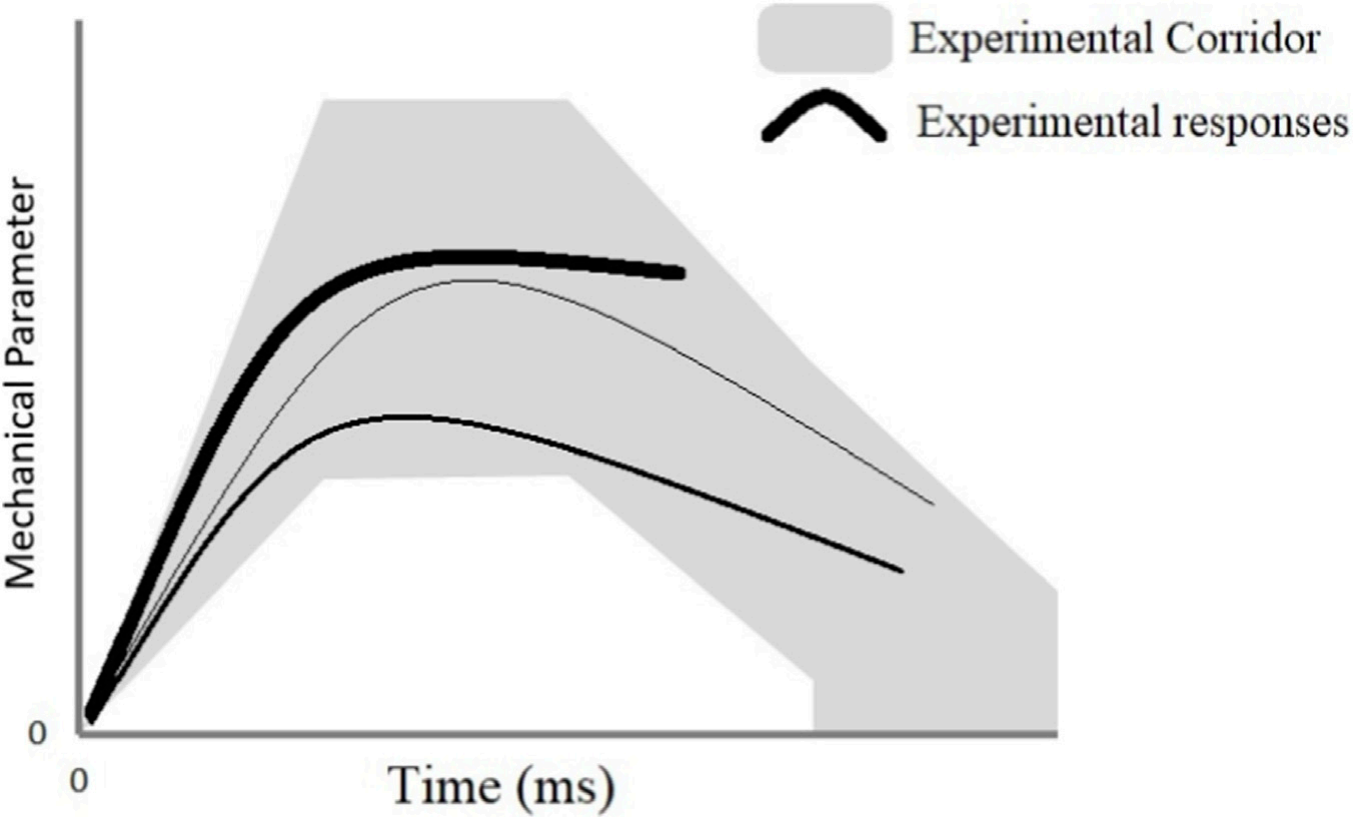
Corridors based on experimental curves defining an inter-individual range of response, based on differences which exist in a given population in terms of anthropometry and in terms of mechanical properties. Adapted from Roth (2023).

However, the corridors are wide, wide enough that models’ response, even when falling within the corridor and thus considered validated, can yield significantly different responses, potentially giving the impression of a “random” outcome as illustrated in Figure 1. In addition, the design of corridors can also be questioned: Indeed, corridors are based on normalized responses which take into account body anthropometry, and scaling technics which can lead to their limitations (Sun et al., 2016).

These limitations do not imply that the corridors are useless or lack scientific validity. They account for the various sources of variability in the human body and are essential for the validation of numerical models. Rather, these limitations are simply factors that must be considered when assessing the biofidelity of biomechanical models and the level of confidence they can inspire.

### 3.3 No validation data: statistical-based analysis and absolute versus relative analysis

The step of validation of a numerical model, in general, lies on a comparison between its response and experimental data. Discrepancies should then be the smallest as possible in order to consider the model as close to the “real world structure”. In the context of biomechanics, numerical models should be validated against existing experimental data to be considered as biofidelic (Roth et al., 2013; Giordano and Kleiven, 2014). Once validated, and once considered as a numerical twin of a real human segment, the numerical model can then be coupled to another model which simulates the environment (Miehling et al., 2024), and in the present context, a protecting structure, in order to assess its capability to mitigate a mechanical loading (a helmet which will protect the head, for example Deck et al., 2003). Results of such a simulation will then provide an “absolute value” of the injury criteria in the biomechanical model, which will then provide information about the protecting ability of the system: if the injury metric is lower than the threshold, the system is efficient. If it is higher, the system does not protect enough. This process can then provide an optimization procedure for the development of the protecting system, which includes biomechanical criteria.

**FIGURE 2:**
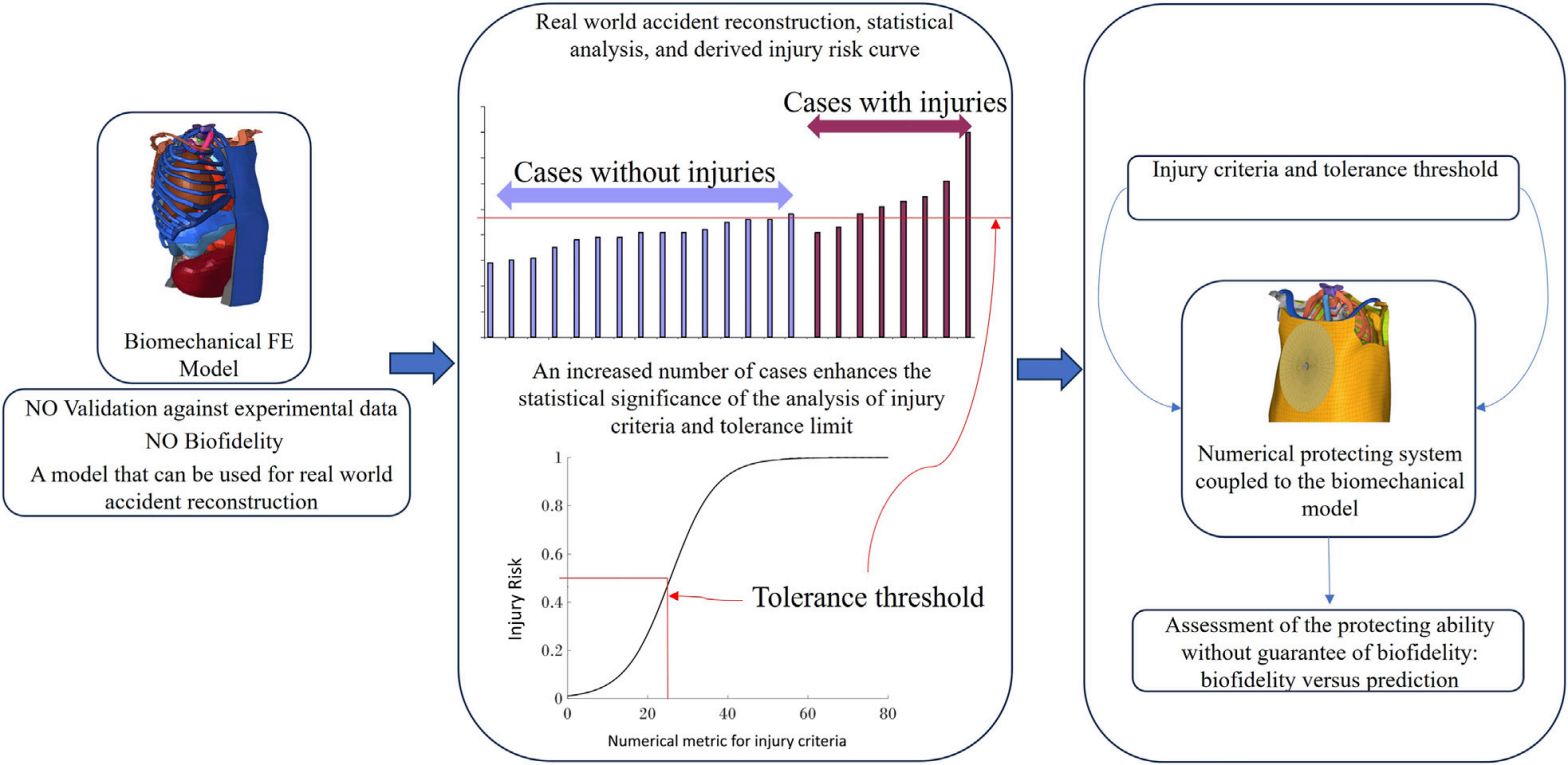
Different way to go further in biomechanics, considering real world cases numerical replication (a significant number number of cases allowing a relevant statistical analysis).

However, as previously explained, there is a lack of experimental data in the literature, which sometimes warn to validate a biomechanical model. It can be noticed for example, studies of literature which deal with child or infant, where experimental data are extremely complex to obtain due to strict ethical rules. In that context, particularly, Roth et al. (2009), explained the difference that could be made between the biofidelic behavior (and then the validation), and the predicting ability. In this study, he explained that the lack of experimental data for validation should not be a brake to the biomechanical analysis of child trauma, and claimed that a statistical analysis on a significant number of numerical recreations of real world data, can provide a statistical-based numerical metric for injury appearance, which can possibly be correlated with a specific injury. This other process can also be applied in biomechanics in order to develop a tool able to predict appearance of lesions even if realism of this tool cannot be ensured by a classical validation process. The following Figure 2 illustrates this point. These previous statements do not aim at minimizing the role and the importance of the validation process; validation being fundamental for model’s trust; It only suggests that some alternatives may be possible if no validation are possible.

This concept of predicting ability without insurance of absolute numerical values, neither biofidelity, can be extended to protecting device. Indeed, in the case of a biomechanical model is designed with an aim of improvement of an existing protecting device, a relative process can be applied: a non-validated biomechanical model is considered, which will be coupled to a protecting existing structure. An optimization process is conducted on the protecting structure with an improvement purpose. A comparative study can then be applied to both systems (biomechanical model with the initial structure and biomechanical model with the optimized structure). Results of both simulations can be compared, and metrics in the biomechanical model can be evaluated: if the biomechanical model coupled with new optimized structure provides lower metrics of the injury criteria than with the first version of the model, the optimized model can be considered as better than the older version, and can be consider as an enhancement since it decreases the value of the injury criteria. Then, this “relative process”, which compare two configurations can lead to an improvement of the protecting structure, without guarantee that the new structure has a realistic mechanical behavior (and without being validated against experimental data).

**FIGURE 3:**
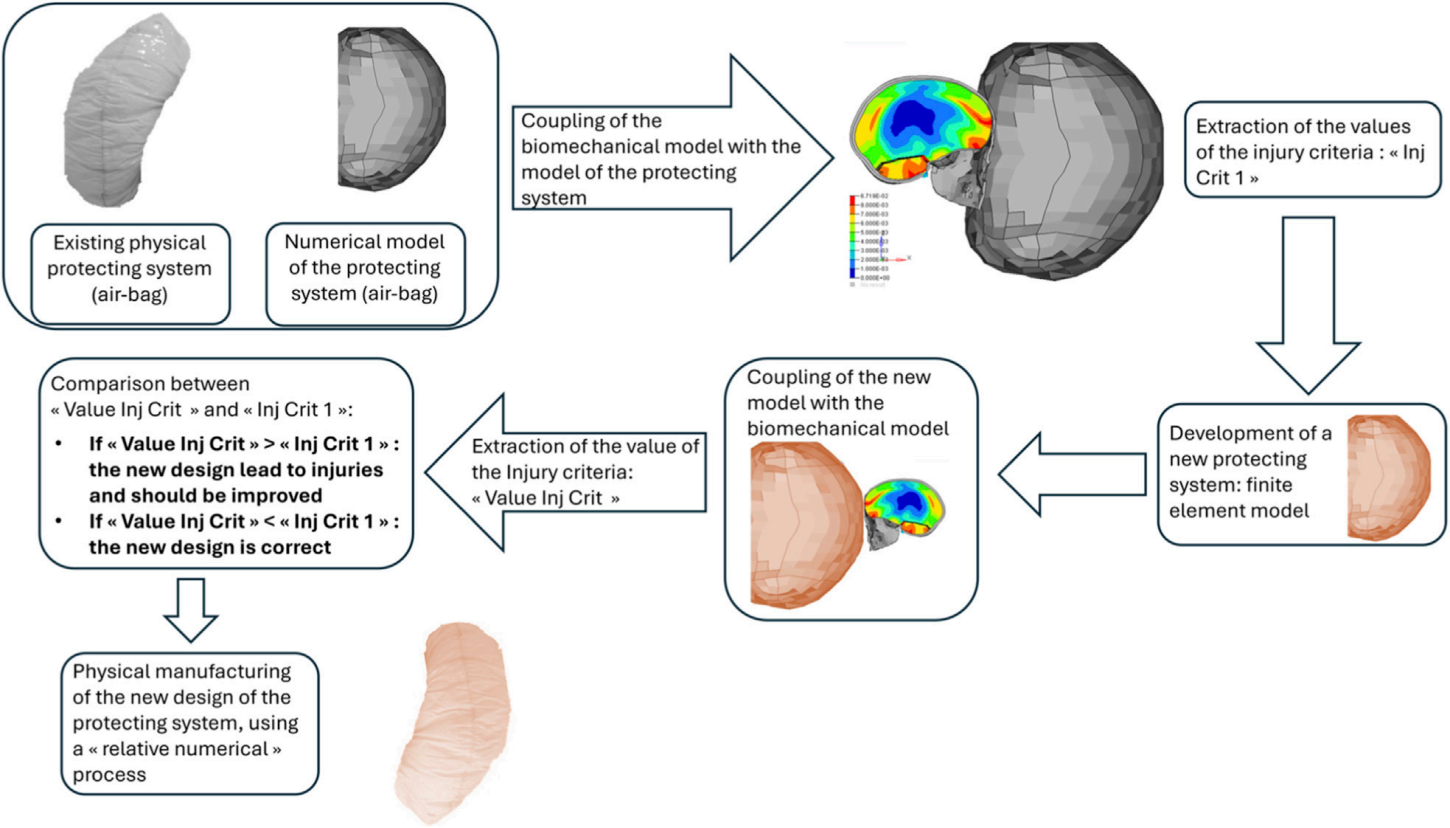
Different way to go further in biomechanics, considering relative process which free from validation of the new concept of protecting device, and based solely on a digital strategy, which allows developing a new protecting device (red air-bag), from an initial simulation using existing protecting device (grey air-bag).

Finally Figure 3 illustrates a way to develop a new concept of protecting device in using only a relative numerical process avoiding experimental tests. Indeed, the process does not focus on the real behavior of the new protecting device, and no validation step is conducted. Only a relative analysis compared to the existing protecting device is performed, without any verifications of the biofidelity of the simulation. The development of the new design is based on an exclusive comparative numerical-based strategy.

Both configurations of simulation (the relative analysis and the statistical-based analysis) previously explained, can provide interesting informations about the behavior of a protecting structure, without checking that the biomechanical model is validated. As previously stated, the lack of experimental data for validation should not be a brake to biomechanical analysis and investigation of protecting structures, and some alternatives should be found to overcome the limitation of validation data. In these cases and in a more global point of view at a numerical level, some workflow should then be used to minimize uncertainties provided by a non-validated model and to reduce the risk of obtaining unrealistic results (benschmarks, model sensitivity, comparison with other codes…) (Roy, 2005; Roy and Oberkampf, 2011; Henninger et al., 2010).

Finally, this section only reports some possible alternative process to explore biomechanical protections under impact, without biomechanical validation data. However, readers should notice that one can not prove a model is valided without physical references, and author does not promote an approach that disregards the validation stage.

### 3.4 Predicting ability versus biofidelity

Ultimately, numerous sources of error may arise in the development of a numerical model representing the human body and its digital twin throughout its lifecycle—from creation, through validation, to its use for the design of surrounding structures. Such models are also subject to biases that may be introduced at different stages. Nevertheless, these biases must remain limited in order to preserve the biofidelity of the studied structures and to allow a meaningful assessment of the predictive capabilities of the models.

It is, however, legitimate to question the entanglement between the notions of biofidelity and predictive capability, since a numerical model may well predict a given behavior (mechanical or biomechanical, in the context of the present work) without being fully biofidelic. For instance, in the case of an impact scenario at the organ level (macroscopic scale), numerical models may exhibit biofidelic behavior at that scale, while failing to do so at smaller scales, as the models implemented in computational codes generally do not account for microscopic phenomena.

**FIGURE 4:**
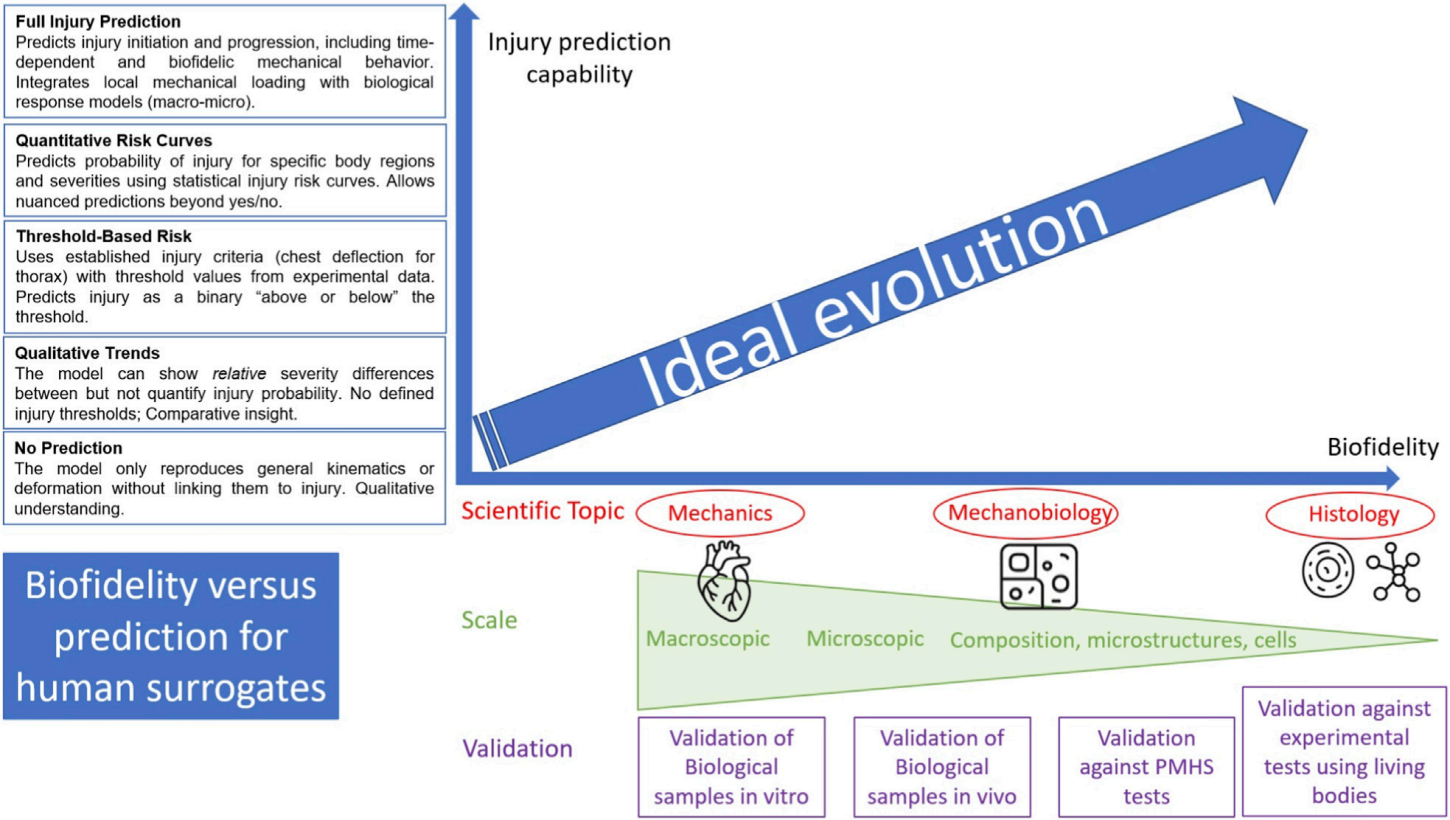
Injury prediction capability versus biofidelity of a model for healthcare, taking into account different scientific topics, different scales, different validation levels, and various qualitative and quantitative parameters for injury prediction. Ideal evolution should take all these concepts into account, for an objective of a complete trust of the digital human.

Predictive capability and biofidelity can therefore be regarded as disjoint concepts, both contributing to the development of digital twins. Trust and acceptance of numerical models thus rely on the advancement of these two notions, which may evolve independently. The ideal evolution of the digital twin is therefore a dual progression of both, as illustrated in Figure 4.

## 4 Toward trust in the use of digital twin: several rigorous ways are possible

### 4.1 Framework and uncertainties in digital biomechanics

Finite element (FE) human body models have emerged as essential tools in the design and evaluation of impact protection systems across automotive, sports, and defense applications. They allow researchers and engineers to investigate biomechanical responses that cannot be directly measured in experiments, to simulate a broad spectrum of loading conditions, and to optimize protective systems while reducing both experimental costs and the ethical issues associated with human or post-mortem testing. Nevertheless, the reliability and credibility of these models within industrial development and regulatory decision-making frameworks are intrinsically linked to how well their underlying uncertainties are characterized and managed. Each component of the modeling process—from anatomical reconstruction and material definition to boundary conditions specifications and numerical implementation—introduces potential sources of variability and error that can significantly influence the model’s predictive performance.

The reliability and societal acceptance of numerical models in impact biomechanics depend on a transparent, scientifically grounded approach to their development and application. Model users should explicitly acknowledge and quantify the uncertainties associated with both mechanical properties and the numerical process itself. Sensitivity analyses and uncertainty quantification must be systematically conducted to assess how input variability influences predicted outcomes. The concepts have already been explored in the literature. It is then of interest to point out Viceconti’s research, who worked on these concepts and highlighted the relevance of biomechanical finite element models. Even if his work was not directly linked to impact biomechanics simulations, he conducted in sillico trials conducting to the validation, the verification and the quantification of uncertainties of biomedical devices (Viceconti et al., 2021; Pappalardo et al., 2022; Taddei et al., 2006; Aldieri et al., 2023). With no doubt, these conclusions can be extended to impact biomechanics, for the application of finite element models in this specific framework. The work is regarded as one of the fondational contribution to the ASME V&V 40 framework (The American Society of Mechanical Engineers, 2018). Clear process is then explained to ensure trust (and then acceptance), of a model at a numerical level. One of these known uncertainties, and which is one of the main limited point in the intrinsic definition of a “model”, concerns the geometry and the anthropometry, and the derived question about how to represent a 50th percentile model of a population, knowing the intra and inter-variability of the human body? How far should simplifications go? Without going until meso or microscopic geometries (biological structures), even macroscopic simplifications can compromise anatomical accuracy and introduce deviations in the predicted mechanical response, particularly in regions characterized by complex contact interfaces such as joints, soft tissues, and internal organs. Complementary to general anthropometry, further uncertainties stems from the mechanical behavior of biological tissues, which are highly variable across individuals and sensitive to factors such as loading rate, strain magnitude, and age- or sex-related changes. Experimental data describing these properties remain scarce and often display significant variability. Thus, the combination of purely numerical parameters (which enable the initiation and solution of the discretized problem), purely mechanical parameters, and finally the geometry (anthropometry) constitutes potential sources of errors that must be kept in mind, as they can affect the results in terms of stress or strain distribution during an impact simulation.

### 4.2 Biofidelity? Predicting capability?

Thus, choices linked to the mesh, the time step, the numerical solver can affect the stability of the calculation, and introduce inaccuracies and purely numerical errors within the model.

In this context, the distinction between biofidelity and predictive capability is of fundamental importance. Biofidelity refers to the ability of a model to accurately reproduce the physical response of the human body as observed experimentally, both in terms of global kinematics and local mechanical quantities such as stress and strain. It is typically assessed through experimental validation procedures, in which model outputs are compared to data obtained from human subjects or from instrumented mechanical surrogates. A model is considered validated when its responses fall within established experimental corridors (Figure 1) or exhibit satisfactory quantitative correlations with experimental measurements. Predictive capability, on the other hand, relates to the model’s ability to anticipate or classify the likelihood of injury occurrence under new loading scenarios, even if the quantitative agreement with experiments is not ensured. Consequently, a model may still be valuable for predicting the onset of injury mechanisms or for comparing performance trends across different configurations, without necessarily achieving full biofidelity. Ideally, a computational human body model would possess both strong biofidelity and robust predictive power (Figure 4); however, in practice, scientific and industrial constraints often necessitate the use of models for which complete experimental validation has not been yet fully achieved.

In cases where experimental validation cannot be conducted, several methodological approaches can still ensure that the model is applied rigorously and responsibly. One approach focuses on sensitivity analysis to determine which parameters most significantly affect the outcomes, allowing experimental efforts to target the most influential quantities. Concurrently, uncertainty quantification can assess the variability in model predictions arising from input parameter uncertainties. Probabilistic techniques, such as Monte Carlo simulations or computational design-of-experiments (Eberle et al., 2018; Marchiori et al., 2019), can then be used to generate a response range rather than a single result, highlighting the model’s robustness or vulnerability to both biological and numerical variability.

In a design framework, a non-validated model can still be useful by employing a comparative or relative strategy. The principle is that if uncertainties influence two simulated configurations in a similar manner, the observed differences between them can serve as reliable indicators of relative performance improvement. Accordingly, the effectiveness of a new protective system can be evaluated against a reference device under the same assumptions and using the same model. In this context, the strength of the analysis depends on internal consistency and reproducibility rather than absolute experimental accuracy. A key step is to assess the robustness of the observed difference, ensuring it remains significant across the entire uncertainty range.

In practice, the use of any numerical model, whether validated or not, must be supported by thorough documentation. This should include a precise account of the geometry, meshing assumptions, constitutive laws and their sources, boundary conditions, contact interfaces, as well as computational and post-processing settings. Model outputs should be reported as distributions (e.g., mean, median, confidence intervals) rather than single deterministic values, with a sensitivity analysis to pinpoint the primary contributors to variability. When design decisions are based on unvalidated injury criteria, implementing safety factors can be an added precautionary measures.

Ultimately, the predictive capability of a model and its biofidelity may represent two orthogonal yet complementary notions that can be combined to achieve an optimal model; ideally, both concepts should be incorporated within the same framework. Even if not validated, a numerical model can still be employed, provided that appropriate precautions, recommendations, and limitations are clearly stated. This approach allows, step by step, for a progressive understanding of injury mechanisms, tolerance limits, and the development of derived protection systems.

### 4.3 Beyond the technical aspects

Beyond technical aspects, transparency and traceability are crucial for the scientific and societal acceptance of numerical models. It is important to clearly communicate to regulators, clinicians, and the public, the model’s confidence level, inherent uncertainties, and underlying assumptions. Any lack of experimental validation must be explicitly acknowledged, with a clear statement of the scope and limitations of the conclusions. Social and regulatory acceptance also relies on developpers engagement to ensure that modeling assumptions are consistent with the physiological and clinical realities of injuries.

Implementing such models requires a strong scientific culture within teams: engineers must be trained in verification, validation, and uncertainty quantification and close collaboration between engineers, clinicians, and biomechanical researchers is essential. Open models, shared datasets, and reproducible analyses are key to building collective trust in these approaches.

Biomechanical numerical models can be valuable for protective system development even in the absence of experimental validation, if a rigorous, transparent, and cautious approach is followed. Non-validated models can be used effectively in comparative emphasizing the robustness of differences of mechanical behavior of protecting system rather than absolute values, or in probabilistic frameworks, where the analysis can bring statistical-based injury metrics for injury occurrence allowing using predicting tool for protection development. Then, some hierarchy of recommendations may be proposed, defining different levels of biofidelity requirements and their corresponding fields of application or corresponding user profiles, and according to available data for validation purpose. This classification aims to provide practical guidance for determining the level of model validation and predictive reliability required, depending on the intended use of the numerical human body model. Inspired by existing classification (ASME V&V 40, FDA, 2021; Groesser and Schwaninger, 2012) four categories are proposed in the following Table 1, in terms of levels (imposed, minimal, acceptable, best), with the corresponding framework, also considering that some overlaps are possible between these categories.

**TABLE 1:** Classification, practical guidance and level of model validation and predictive reliability.

Level	Framework and user	Objective	Validation & biofidelity requirements	Use scope
Imposed	Regulatory, certification, standards, production	Decision-making with safety or legal implications	Full experimental validation, traceable documentation, uncertainty quantification	Implementation/production
Best practice	Scientific research	Advancing the state of knowledge in biomechanics, establishing new injury criteria, or supporting standards development	Multi-scale validation, probabilistic modeling, reproducibility	Fundamental/advanced research
Minimal	Early R&D, concepts and exploration	Early design screening, trend analysis, or hypothesis testing	Numerical verification only, qualitative plausibility checks	Exploratory/relative comparison
Acceptable	Applied R&D, pre-certification	Design improvement, performance ranking, or correlation with limited experimental data	Partial experimental validation, sensitivity & UQ, defined applicability	Development/optimization

This hierarchical approach acknowledges that a universal or fixed level of biofidelity cannot be prescribed for all applications. The degree of validation required should be determined by the level of risk linked to the decision-making context, the extent and quality of available experimental data, and the specific purpose for which the model is employed. In scenarios involving critical safety assessments or regulatory certification, comprehensive validation supported by quantified uncertainty analysis remains important. In contrast, for exploratory, conceptual, or comparative studies, models with limited biofidelity can still provide significant insight, as long as their inherent limitations are clearly documented and interpretations are confined to relative performance trends rather than absolute injury metrics. Implementing such a hierarchical framework promotes greater transparency, reproducibility, and accountability in model use, while simultaneously driving the field toward progressively higher standards of validation and predictive reliability.

## 5 Conclusion

Numerical modelling in biomechanics and related digital surrogates for healthcare have lots of benefits for industrials or for medical purpose. Their biofidelity and predicting ability can provide very interesting data to enhance human protection in the context of impact biomechanics. The implicit complexity of the development of such digital humans raises questions about trust and acceptability of their use. It is then of interest to have a complete understanding of their creation, to be aware about their limitations, and to use them within a well-defined context, thereby enabling informed design choices, and to promote their use to the public in order to ensure indisputable applicability and acceptability.
